# Supplementary material for: A hexokinase isoenzyme switch in human liver cancer cells promotes lipogenesis and enhances innate immunity
Source: Commun Biol. 2021 Feb 16;4:217. doi: 10.1038/s42003-021-01749-3 (PMC7886870; doi:10.1038/s42003-021-01749-3)
Supplement: Supplementary file 2 — Supplementary Information [file 42003_2021_1749_MOESM2_ESM.pdf]

A hexokinase isoenzyme switch in human liver cancer cells promotes lipogenesis and enhances innate immunity

Laure Perrin-Cocon[https://orcid.org/0000-0003-0501-6733,1,†], Pierre-Olivier Vidalain[https://orcid.org/0000-0003-3082-7407,1,†], Clémence Jacquemin[1], Anne Aublin-Gex[1], Keedrian Olmstead[2], Baptiste Panthu[1,3], Gilles Jeans Philippe Rautureau[4], Patrice André[1], Piotr Nyczka[5], Marc-Thorsten Hütt[5], Nivea Amoedo[6], Rodrigue Rossignol[6,7], Fabian Volker Filipp[http://orcid.org/0000-0001-9889-5727 2,8], Vincent Lotteau[http://orcid.org/0000-0003-0997-3282,1,*‡] Olivier Diaz[https://orcid.org/0000-0002-3451-9914,1,*‡]

1- CIRI, Centre International de Recherche en Infectiologie, Univ Lyon, Inserm, U1111, Université Claude Bernard Lyon 1, CNRS, UMR5308, ENS de Lyon, 21 Avenue Tony Garnier, F-69007, Lyon, France.
2- Cancer Systems Biology, Institute for Diabetes and Cancer, Helmholtz Zentrum München, Ingolstädter Landstraße 1, D-85764, München, Germany.
3- Actual address: Univ Lyon, CarMeN Laboratory, Inserm, INRA, INSA Lyon, Université Claude Bernard Lyon 1, Hôpital Lyon Sud, Bâtiment CENS ELI-2D, 165 Chemin du grand Revoyet F-69310 Pierre-Bénite, France.
4- Université de Lyon, CNRS, Université Claude Bernard Lyon 1, ENS de Lyon, Centre de RMN à Très Hauts Champs (CRMN), FRE 2034, 5 rue de la Doua, F-69100 Villeurbanne, France.
5- Department of Life Sciences and Chemistry, Jacobs University, Campus Ring 1, D-28759 Bremen, Germany.
6- CELLOMET, Centre de Génomique Fonctionnelle de Bordeaux, 146 Rue Léo Saignat, F-33000 Bordeaux, France.
7- Univ. Bordeaux, Inserm U1211, MRGM, Centre hospitalier universitaire Pellegrin, place Amélie Raba Léon, F-33076, Bordeaux, France.
8- School of Life Sciences Weihenstephan, Technical University München, Maximus-von-Imhof-Forum 3, D-85354, Freising, Germany.

† These authors contributed equally to this work
‡These authors jointly supervised this work

*** Corresponding authors:**
vincent.lotteau@inserm.fr (ORCID: 0000-0003-0997-3282)
olivier.diaz@inserm.fr (ORCID: 0000-0002-3451-9914)

Keywords
Hepatocellular carcinoma, hexokinase, lipogenesis, innate immunity, lipoproteins.

This PDF file includes:
Supplementary Figures

Supplementary figure 1

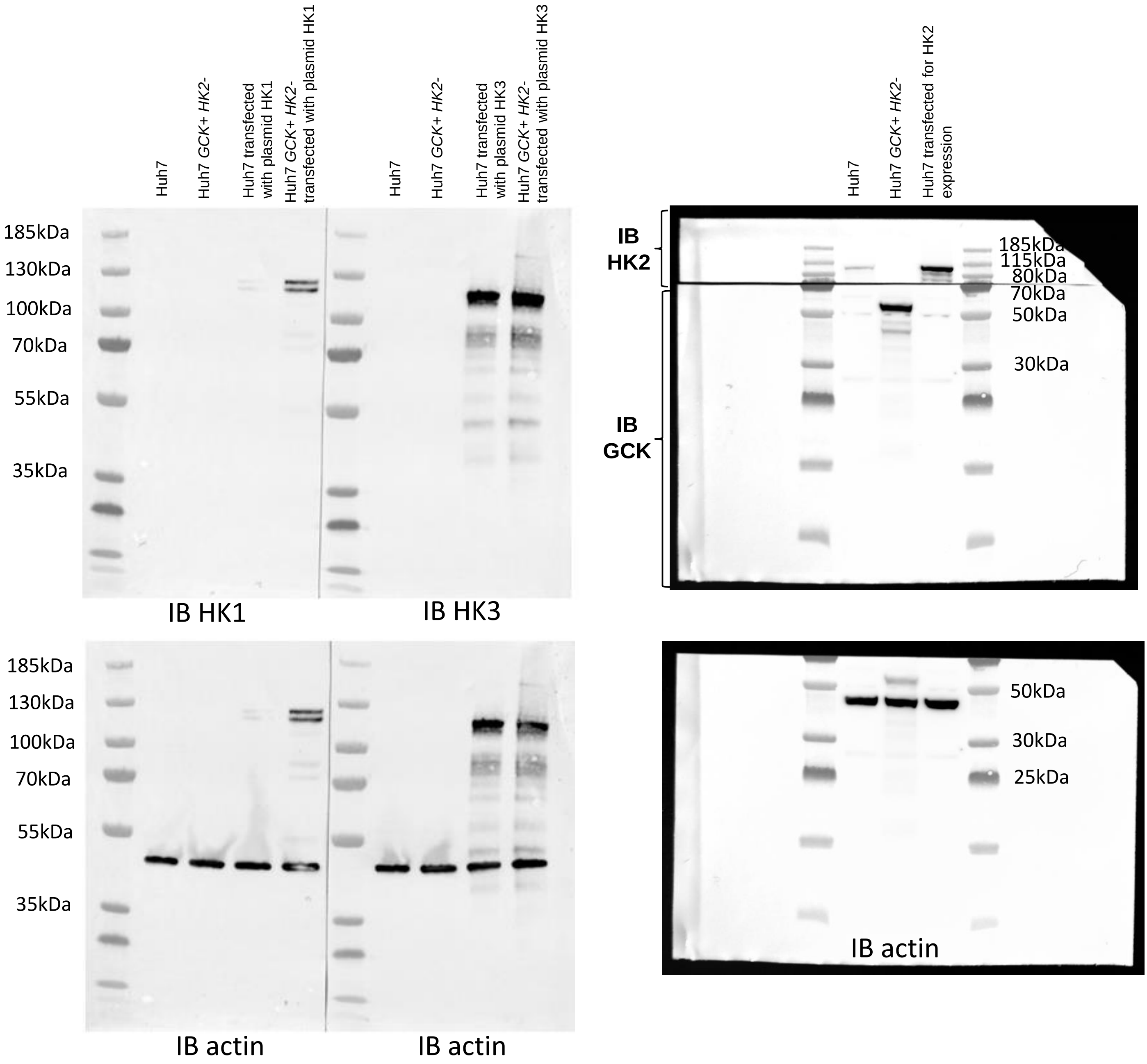

Supplementary Figure 1: Uncropped membranes of western-blotting presented in Figure 2a. Immunoblot (IB) of HK1, HK3, HK2 and GCK. IB for actin was performed on the same membrane for normalization. On the right panel, the PVDF membrane obtained after protein transfer was cut in two fragments above and below 70kDa. The upper membrane fragment was incubated with the anti-HK2 antibody, whereas the lower membrane fragment was incubated with the anti-GCK antibody.

Supplementary Figure 2

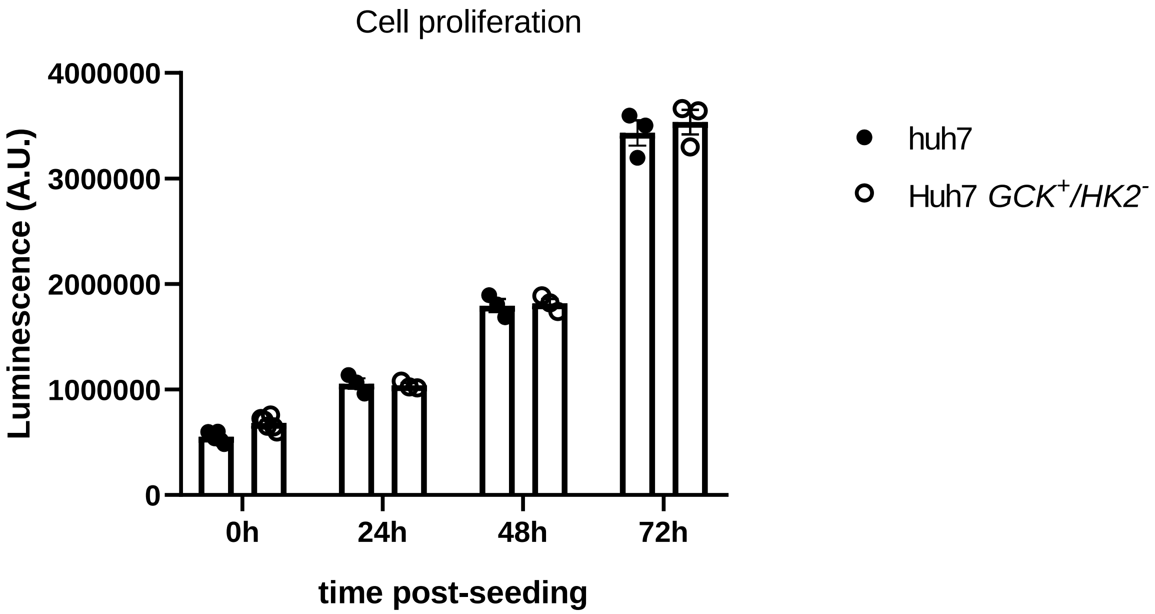

Supplementary Figure 2. Proliferation of Huh7 and Huh7-*GCK*$^{+}$*/HK2*$^{-}$. Cells were seeded in 96 well-plate under standard growth conditions and cellular proliferation was determined at time 0, 24, 48 and 72h post-seeding using the CellTiter-Glo® Luminescent Cell Viability Assay (Promega). Luminescence was quantified with an Infinite M200 microplate reader (TECAN). Means ± SEM are presented (n=3).

Supplementary Figure 3

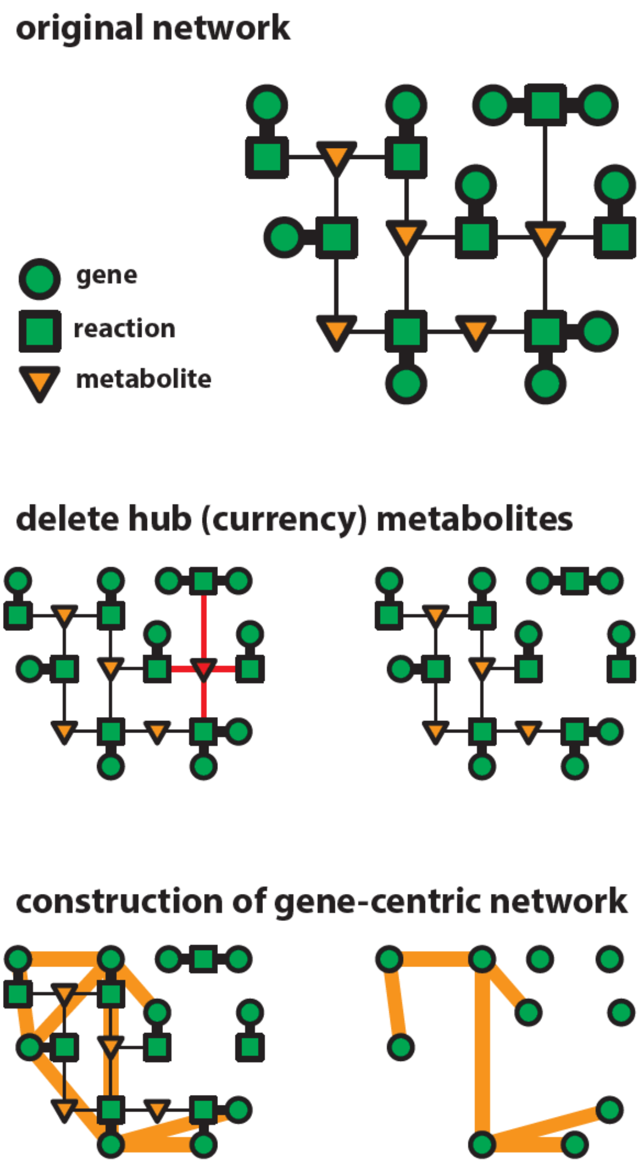

Supplementary Figure 3. Strategy to generate a gene-centric metabolic network from a given genome-scale metabolic network. The original network was extracted from a genome-scale metabolic model where metabolites are connected by reactions that are catalyzed by enzymes. The high-degree nodes ('currency metabolites' like H_2O, ATP, etc.) which are not informative about the network organization of the metabolic systems were eliminated before interpreting the network architecture. The removal of different percentages of currency metabolites was tested to obtain reaction-centric graphs. Then gene-reaction associations were used to generate a gene-centric metabolic network with the highest connectivity (see Supplementary Figure 4).

Supplementary Figure 4

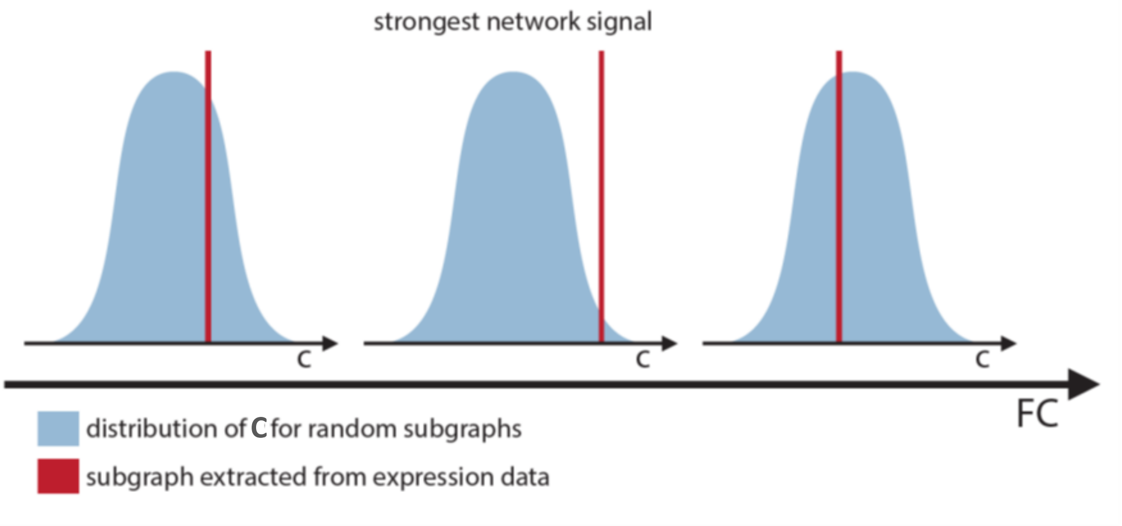

Supplementary Figure 4. Determination of the optimal Metabolic network Coherence (MC). Given a set S of differentially expressed genes and the gene-centric metabolic network G, we analyzed the subgraph of G spanned by all genes in S. The average clustering coefficient C of connected nodes in these subgraphs serves as a measure of the connectivity of this subgraph (see supplementary Figure 5). Different FC-expression thresholds of S were applied to calculate C. The FC-expression threshold providing the highest difference of C in comparison to clustering coefficient obtained for a random subgraph (i.e. giving the strongest network signal) was set to calculate the metabolic network coherence MC presented in Supplementary figure 5).

Supplementary Figure 5

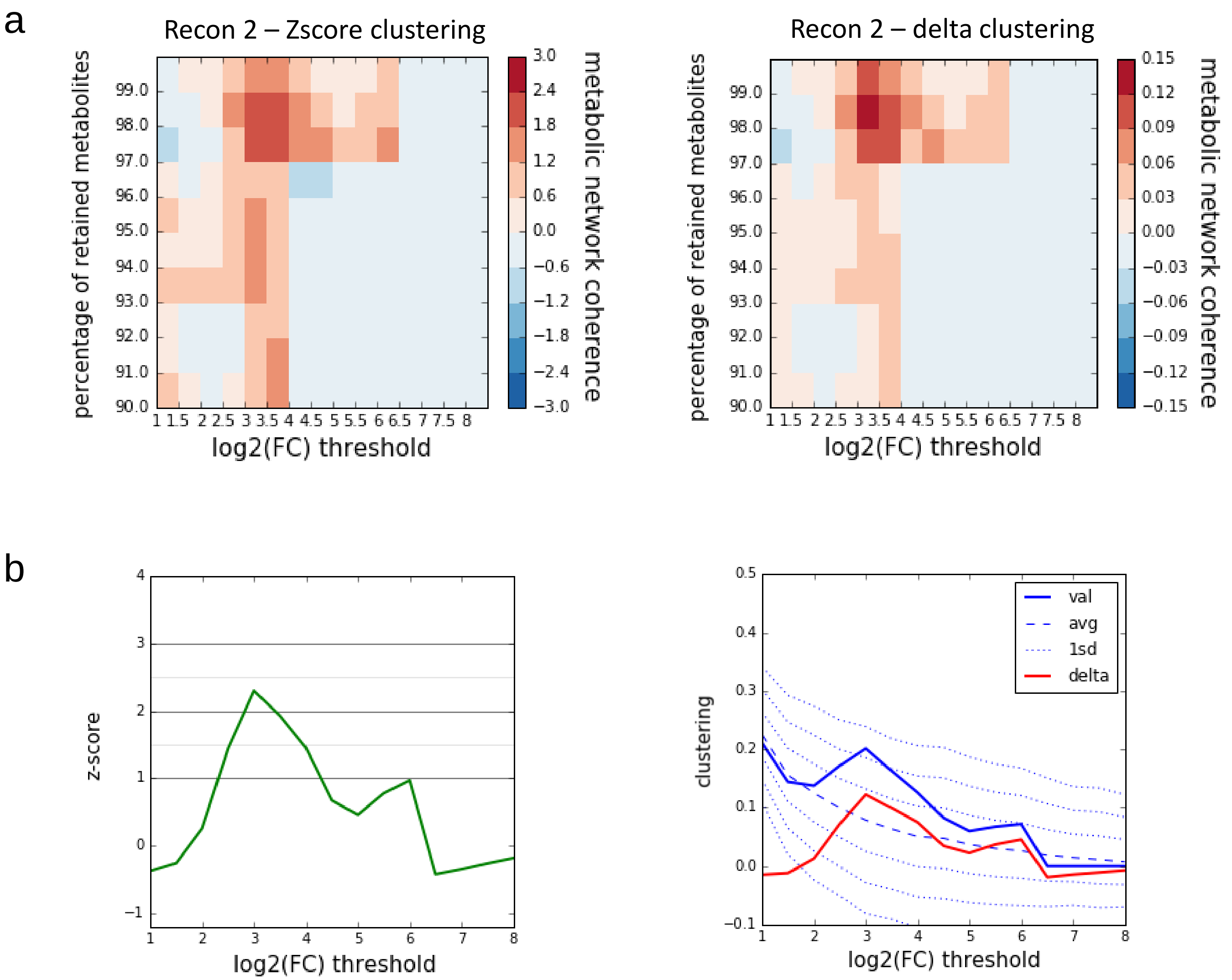

Supplementary Figure 5. Clustering parameters of the constructed metabolic network obtained from the observed gene set S. a Heatmap of Z-scores (left panel) and Delta-scores (right panel) for clustering coefficients obtained when mapping differentially expressed genes to human metabolic model Recon2. Values range from highest in red to lowest in blue as a function of the percentage of retained metabolites (after removal of currency metabolites) and the Fold-Change threshold. **b** Z-score (left panel) and clustering coefficient (right panel) obtained for a currency of 98% (2% of the most connected metabolites removed). Highest Z-score and clustering coefficient C are obtained at log_2(FC) threshold >3. This optimal fold-change threshold was used to construct the network presented in figure 2e.

Supplementary Figure 6

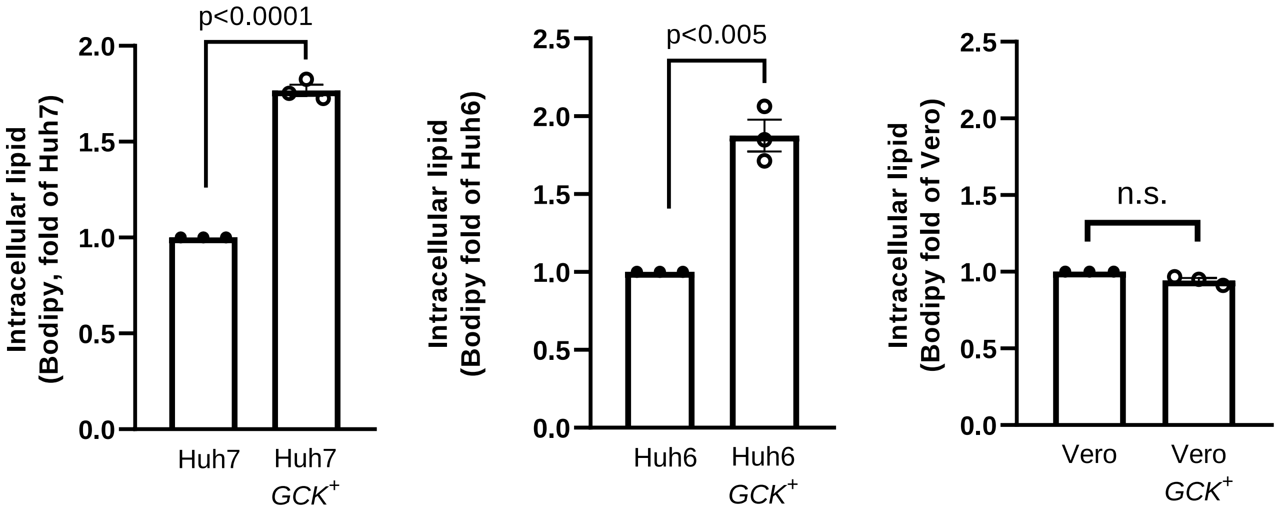

Supplementary Figure 6. Effect of GCK expression on intracellular lipid content of Huh7, Huh6 and Vero cells. Intracellular lipid content of two hepatic and one renal cell lines was analyzed after stable re-expression of GCK. Parental Huh6, Huh7 or Vero cells were transduced with lentiviruses for GCK expression using a pLEX-GCK construct as described in material and methods. Cells were then cultured for 7 days in the presence of puromycin to select transduced cells before amplification. Cells were stained for their intracellular lipid content using BODIPY 493/503 dye and analyzed by flow-cytometry. Means ± SEM (n=3) of fluorescence normalized to the corresponding parental cell line are presented. *p* values were determined using Student's t-test.

Supplementary figure 7

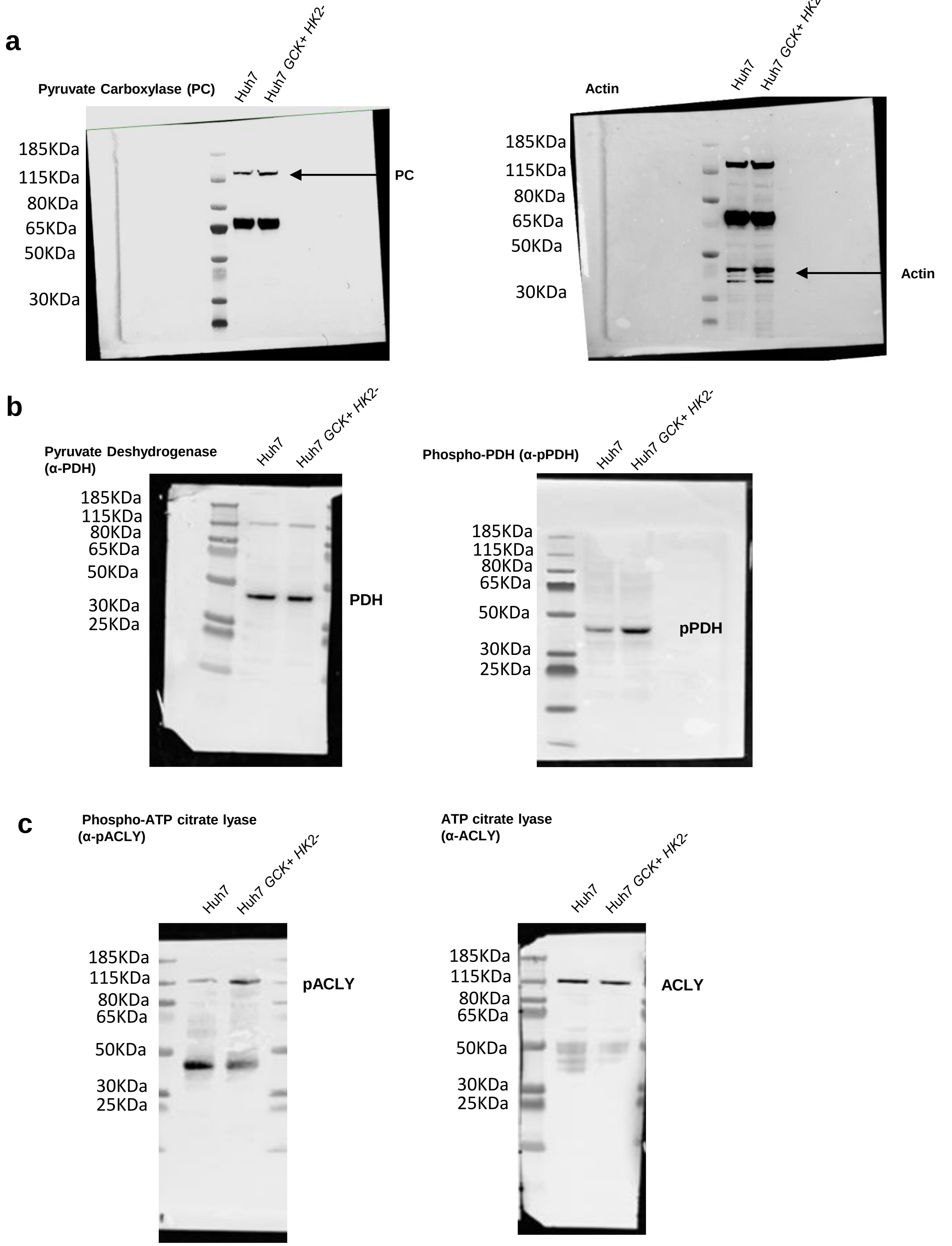

Supplementary figure 7: Uncropped membranes of western-blotting presented in figure 5. a WB of Pyruvate Carboxylase (PC) presented in figure 5g. **b** WB of Pyruvate Deshydrogenase (PDH) presented in figure 5h. **c** WB of ATP-citrate lyase (ACLY) presented in figure 5j.

Supplementary Figure 8

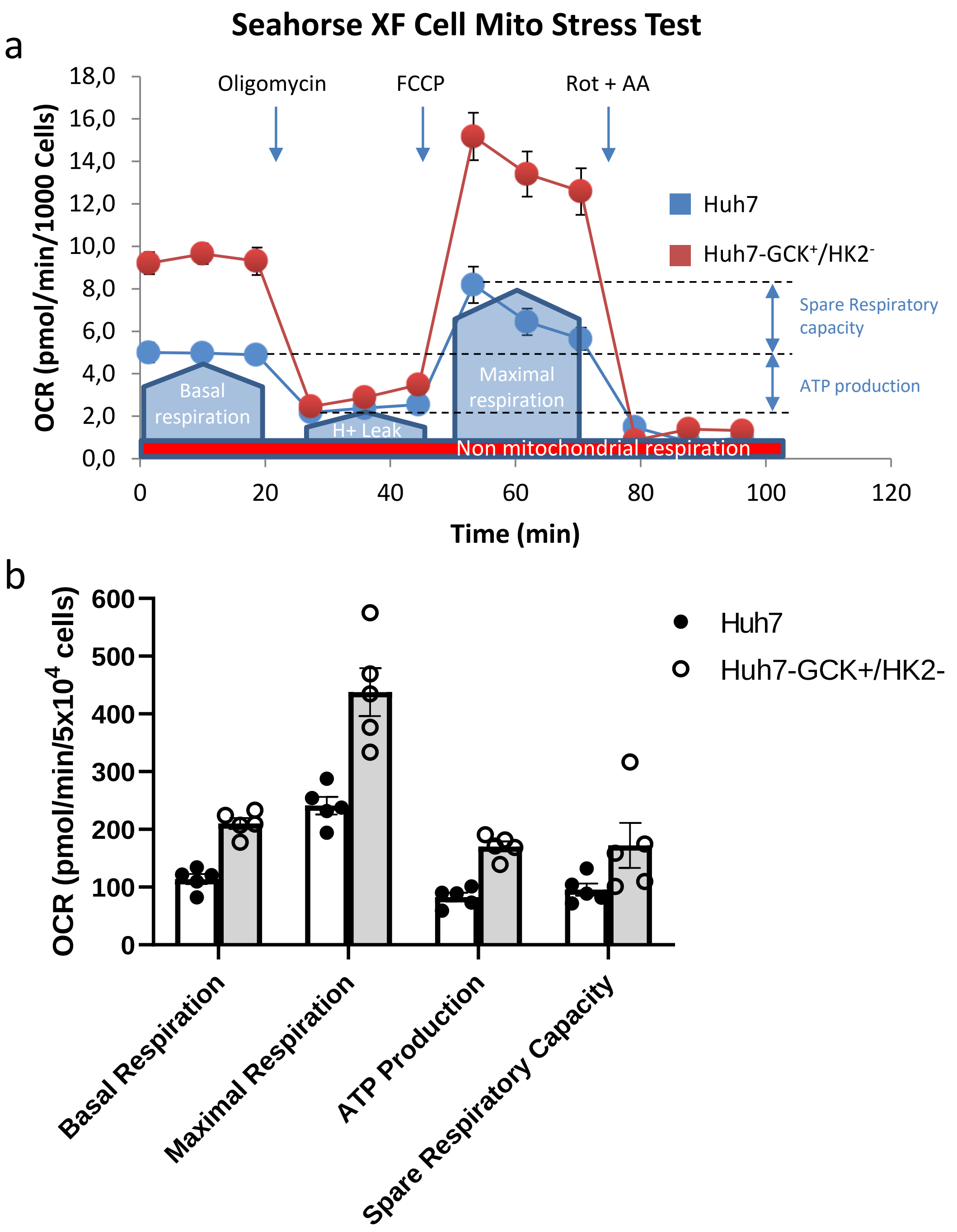

Supplementary Figure 8. Mitochondrial activity parameters a Schematic representation of how basal respiration, maximal Respiration, ATP production and spare respiration capacity are calculated on an example of Oxygen Consumption Rate (OCR) performed with Seahorse XF cell Mito Stress Test. **b** Results presented as individual data with means ± SEM (n=5 biologically independent samples).

Supplementary Figure 9

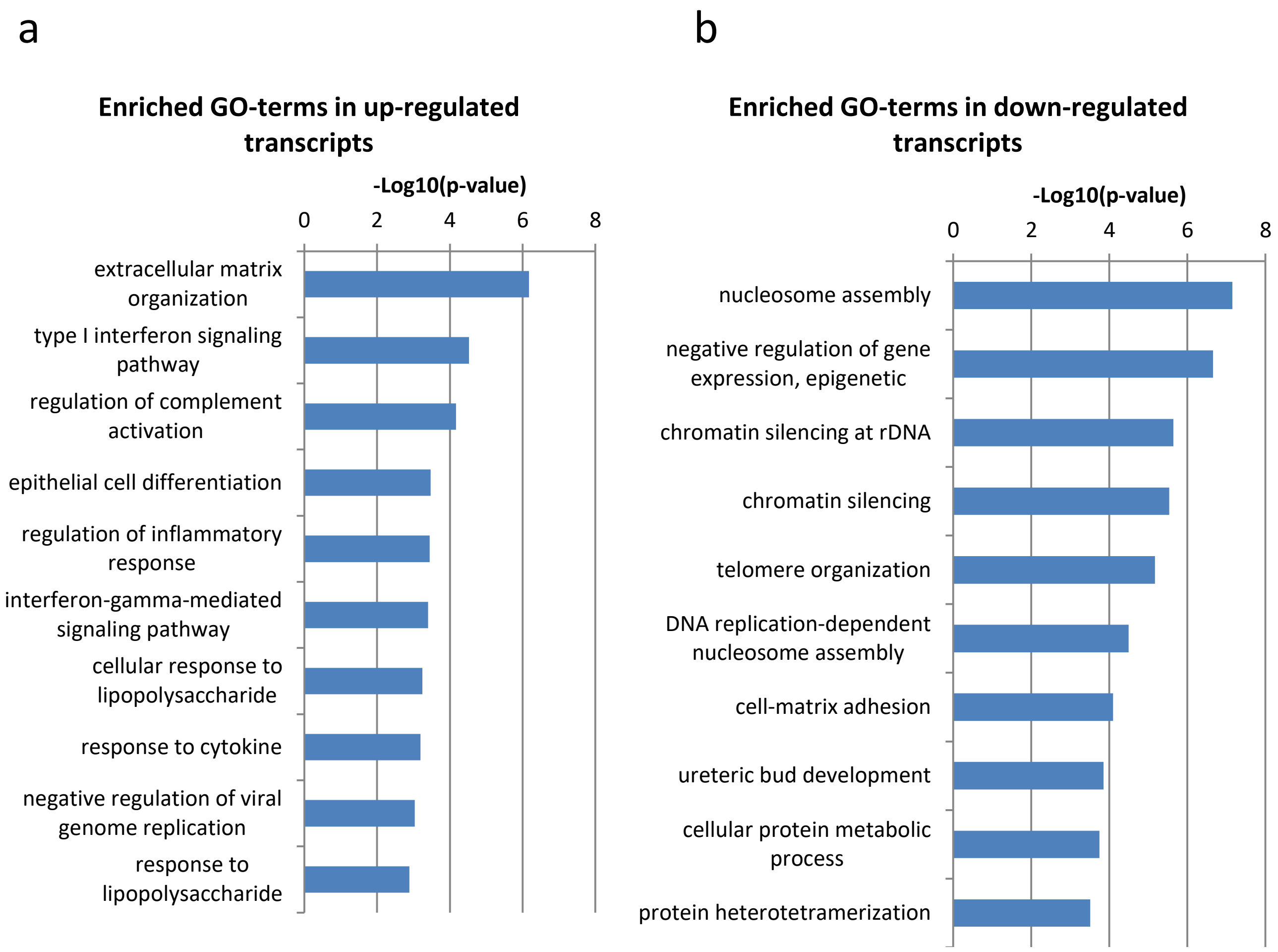

Supplementary Figure 9. Gene Ontology terms enriched in differentially expressed transcripts. GO enrichment analysis of transcripts significantly up- (**a**) and down- (**b**) regulated in Huh7-GCK+/HK2- vs Huh7 cells determined by DESeq2 analysis of transcriptomic data. Transcripts lists were submitted to the Functional Annotation Tool of the online knowledge base DAVID Bioinformatics Resources 6.8, NIAID/NIH. In DAVID, Fisher's Exact test is adopted to measure the gene-enrichment in annotation terms. Parameters were adjusted to a minimal count belonging to the annotation of 10 and EASE score (one-tail Fisher Exact Probability Value) threshold of 0.01.

Supplementary Figure 10

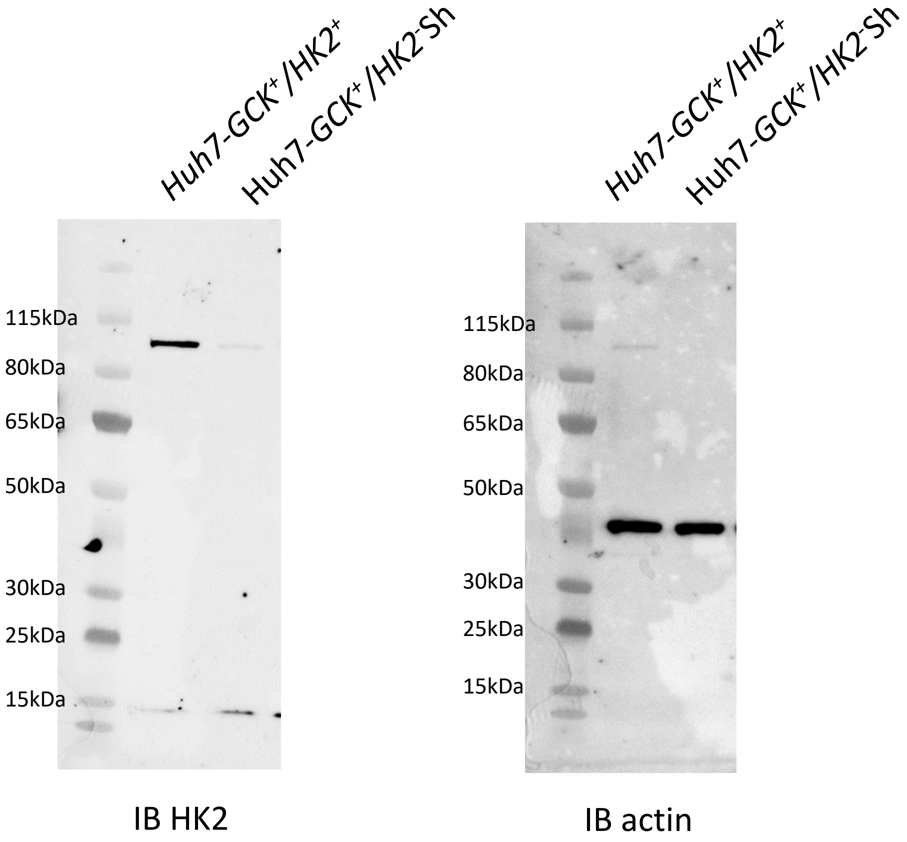

Supplementary Figure 10. HK2 protein extinction by shRNA. Western Blot analysis of HK2 protein expression in Huh7 cells transduced for *GCK* expression before (Huh7-*GCK*$^{+}$/*HK2*$^{+}$) and after extinction by ShRNA (*Huh7-GCK*$^{+}$/*HK2*$^{-}$Sh). Uncropped immunoblot (IB) of HK2 and actin performed on the same membrane for normalization.

Supplementary Figure 11

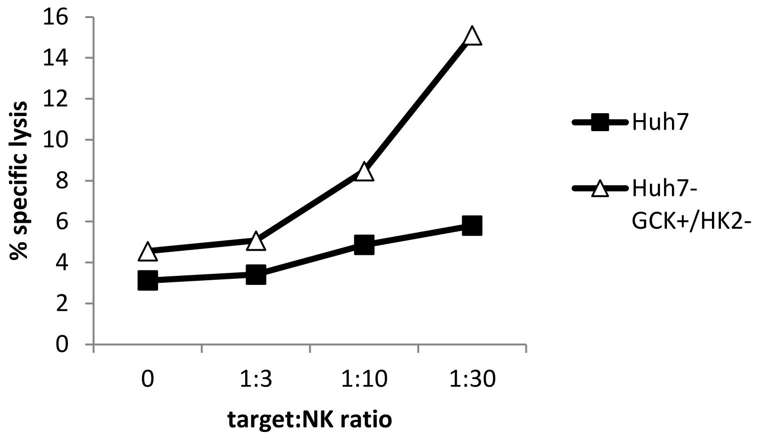

Supplementary Figure 11. Natural Killer (NK) cells sensitivity of Huh7 and Huh7-*GCK*$^{+}$/*HK2*$^{-}$ cells. a NK cell mediated lysis of Huh7 or Huh7-*GCK*$^{+}$/*HK2*$^{-}$. Hepatoma cells were seeded 24 h before addition of IL2-preactivated NK cells for 4 h at effector to target (E:T) ratio of 0, 3, 10 or 30. After harvesting, cells were stained with propidium iodide (PI) and analyzed by flow cytometry. Lysis was determined by the percentage of PI^{+} cells on gated hepatocytes (One representative experiment).

Supplementary Figure 12

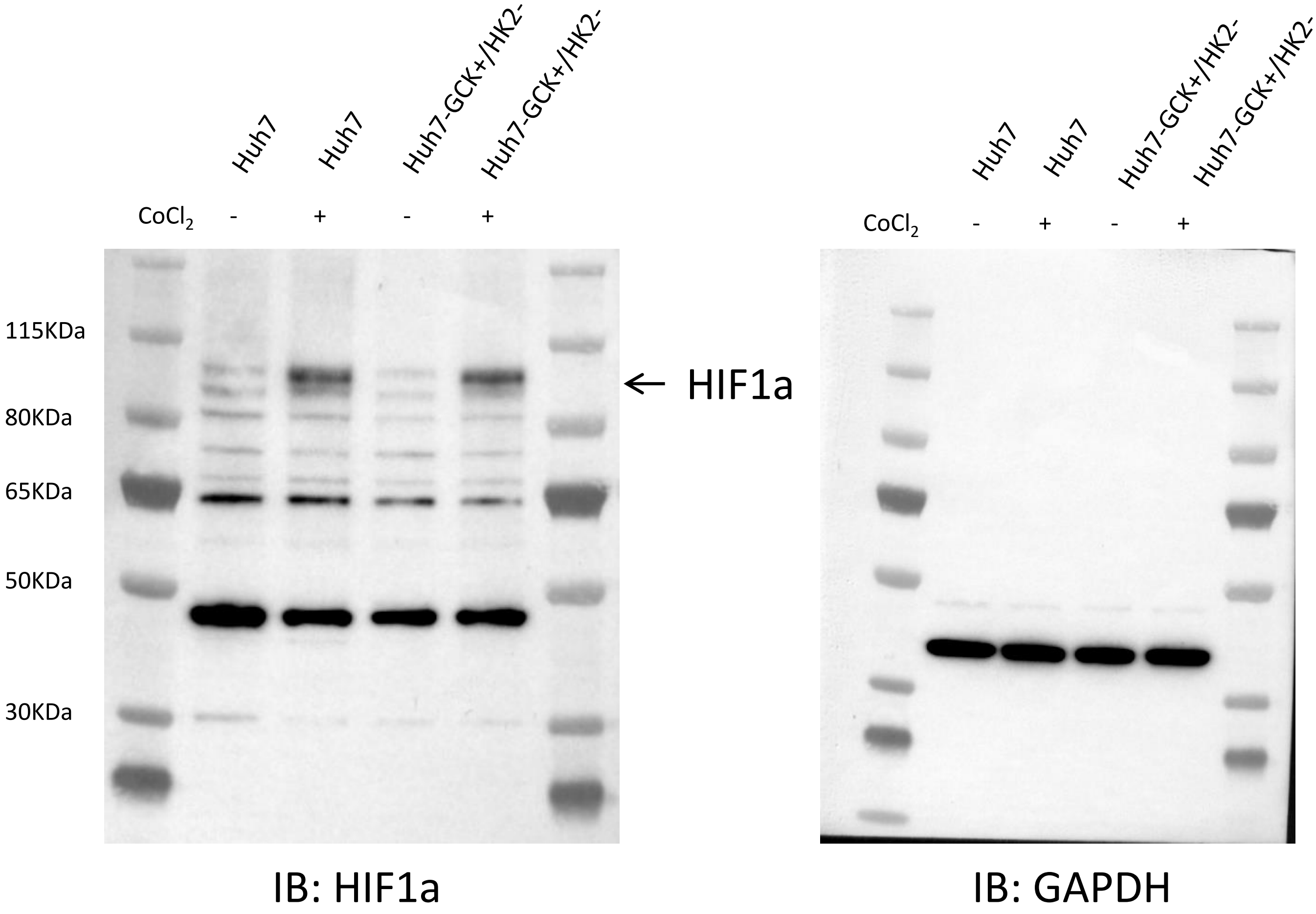

Supplementary Figure 12. **HIF-1α detection.** Western Blot analysis of HIF-1α in Huh7 and Huh7-*GCK*$^{+}$/*HK2*$^{-}$ cells with or without 150 µM $CoCl_2$ treatment to mimic hypoxic conditions. Uncropped immunoblot (IB) of HIF-1a and GAPDH performed on the same membrane for normalization.

Supplementary Figure 13

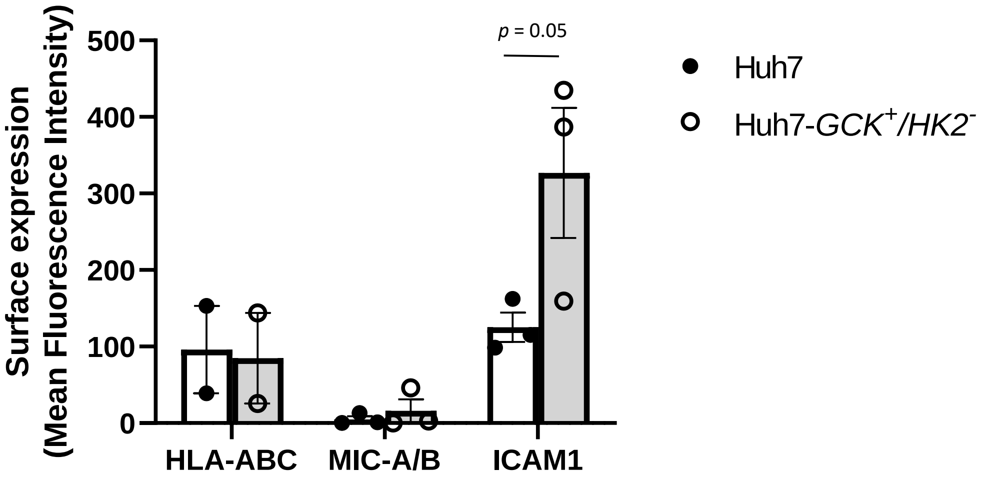

Supplementary Figure 13. Natural Killer (NK) target molecule expression of Huh7 and Huh7-*GCK*$^{+}$*/HK2*$^{-}$ cells. Surface expression of HLA-A,B,C, MIC-A/B and ICAM-1 on Huh7 and Huh7-*GCK*$^{+}$*/HK2*$^{-}$ cells analyzed by Flow cytometry after cell surface immunolabelling with anti-HLA-ABC-PE (BD Biosciences), anti-MIC-A/B-PE (BD Biosciences) or anti-ICAM1-FITC (Beckman Coulter). n=2 or 3 independent experiments, Student's t test.
