# Supplementary material for: A hexokinase isoenzyme switch in human liver cancer cells promotes lipogenesis and enhances innate immunity
Source: Commun Biol. 2021 Feb 16;4:217. doi: 10.1038/s42003-021-01749-3 (PMC7886870; doi:10.1038/s42003-021-01749-3)
Supplement: Supplementary file 3 — Description of Additional Supplementary Files [file 42003_2021_1749_MOESM3_ESM.pdf]

**Description of Additional Supplementary Files**

**File name:** Supplementary Data 1

**Description:** RNA-seq data of 365 HCC biopsies from The Cancer Genome Atlas (TCGA) database. Related to Figure 1.

**File name:** Supplementary Data 2

**Description:** Transcriptomic profiles of Huh7 and Huh7-*GCK*$^{+}$/*HK2*$^{-}$ cells.

**File name:** Supplementary Data 3

**Description:** Gene set enrichment data from Ingenuity Pathway Analysis (IPA).

**File name:** Supplementary Data 4

**Description:** Source data for main and supplementary figures.
