# Supplementary material for: A hexokinase isoenzyme switch in human liver cancer cells promotes lipogenesis and enhances innate immunity
Source: Commun Biol. 2021 Feb 16;4:217. doi: 10.1038/s42003-021-01749-3 (PMC7886870; doi:10.1038/s42003-021-01749-3)
Supplement: Supplementary file 8 — Reporting Summary [file 42003_2021_1749_MOESM8_ESM.pdf]

nature research

Corresponding author(s): Vincent Lotteau, Olivier Diaz

Last updated by author(s): Oct 16, 2020

# Reporting Summary

Nature Research wishes to improve the reproducibility of the work that we publish. This form provides structure for consistency and transparency in reporting. For further information on Nature Research policies, see our Editorial Policies and the Editorial Policy Checklist.

## Statistics

For all statistical analyses, confirm that the following items are present in the figure legend, table legend, main text, or Methods section.

| n/a | Confirmed | |
|---|---|---|
| ☐ | ☒ | The exact sample size (*n*) for each experimental group/condition, given as a discrete number and unit of measurement |
| ☐ | ☒ | A statement on whether measurements were taken from distinct samples or whether the same sample was measured repeatedly |
| ☐ | ☒ | The statistical test(s) used AND whether they are one- or two-sided<br>*Only common tests should be described solely by name; describe more complex techniques in the Methods section.* |
| ☐ | ☒ | A description of all covariates tested |
| ☐ | ☒ | A description of any assumptions or corrections, such as tests of normality and adjustment for multiple comparisons |
| ☐ | ☒ | A full description of the statistical parameters including central tendency (e.g. means) or other basic estimates (e.g. regression coefficient) AND variation (e.g. standard deviation) or associated estimates of uncertainty (e.g. confidence intervals) |
| ☐ | ☒ | For null hypothesis testing, the test statistic (e.g. *F*, *t*, *r*) with confidence intervals, effect sizes, degrees of freedom and *P* value noted<br>*Give P values as exact values whenever suitable.* |
| ☒ | ☐ | For Bayesian analysis, information on the choice of priors and Markov chain Monte Carlo settings |
| ☒ | ☐ | For hierarchical and complex designs, identification of the appropriate level for tests and full reporting of outcomes |
| ☒ | ☐ | Estimates of effect sizes (e.g. Cohen's *d*, Pearson's *r*), indicating how they were calculated |

*Our web collection on statistics for biologists contains articles on many of the points above.*

## Software and code

Policy information about availability of computer code

| | |
|---|---|
| Data collection | no software was used |
| Data analysis | For metabolomics data processing, metabolites were identified using TraceFinder (v3.3, Thermo Fisher Scientific) based on libraries of metabolite retention times and fragmentation patterns (Metaflux, Merced, CA).<br>Gene set enrichment analysis was performed with Ingenuity Pathway Analysis (IPA, Build version: 486617M, Qiagen) |

For manuscripts utilizing custom algorithms or software that are central to the research but not yet described in published literature, software must be made available to editors and reviewers. We strongly encourage code deposition in a community repository (e.g. GitHub). See the Nature Research guidelines for submitting code & software for further information.

## Data

Policy information about availability of data

All manuscripts must include a data availability statement. This statement should provide the following information, where applicable:
- Accession codes, unique identifiers, or web links for publicly available datasets
- A list of figures that have associated raw data
- A description of any restrictions on data availability

The data generated or analysed during this study are included in the article and supplementary files. Entire raw data of transcritomic analysis are deposited in Gene Expression Omnibus database with the accession number GSE144214.

# Field-specific reporting

Please select the one below that is the best fit for your research. If you are not sure, read the appropriate sections before making your selection.

☒ Life sciences ☐ Behavioural & social sciences ☐ Ecological, evolutionary & environmental sciences

For a reference copy of the document with all sections, see nature.com/documents/nr-reporting-summary-flat.pdf

# Life sciences study design

All studies must disclose on these points even when the disclosure is negative.

| | |
|---|---|
| Sample size | Statistical analysis of the results validated sample size |
| Data exclusions | No data were excluded from validated experiments with positive and negative controls |
| Replication | Experiments were reproduced at least 3 times independently. |
| Randomization | For each independent experiment, the treatments on each cell type are carried out on the same number of wells, seeded identically and randomly assigned. |
| Blinding | Analyzes are carried out systematically, regardless of the identity of the samples and their treatment |

# Reporting for specific materials, systems and methods

We require information from authors about some types of materials, experimental systems and methods used in many studies. Here, indicate whether each material, system or method listed is relevant to your study. If you are not sure if a list item applies to your research, read the appropriate section before selecting a response.

## Materials & experimental systems

| n/a | Involved in the study | |
|---|---|---|
| ☐ | ☒ | Antibodies |
| ☐ | ☒ | Eukaryotic cell lines |
| ☒ | ☐ | Palaeontology and archaeology |
| ☒ | ☐ | Animals and other organisms |
| ☒ | ☐ | Human research participants |
| ☒ | ☐ | Clinical data |
| ☒ | ☐ | Dual use research of concern |

## Methods

| n/a | Involved in the study | |
|---|---|---|
| ☒ | ☐ | ChIP-seq |
| ☒ | ☐ | Flow cytometry |
| ☒ | ☐ | MRI-based neuroimaging |

## Antibodies

Antibodies used

mouse monoclonal antibody against human GCK (clone G-6, Santa Cruz Biotechnology #sc-17819),
rabbit monoclonal antibody against human HK2 (Clone C64G5, Cell Signaling Technology #2867),
rabbit monoclonal antibody against human HK1 (C35C4, Cell Signaling #2024),
rabbit polyclonal antibody against human HK3 (Millipore Sigma-Aldrich #HPA056743),
goat polyclonal antibody against human ACLY (Santa Cruz Biotechnology, #sc-30537),
rabbit polyclonal antibody against human pACLY (phospho S455, Cell Signaling Technology #4131),
rabbit monoclonal antibody against human PDH (C54G1, Cell Signaling Technology #3205),
rabbit monoclonal antibody against human pPDH E1-alpha subunit (phospho S293, Abcam #ab92696),
goat polyclonal antibody against human PC (Millipore Sigma-Aldrich, #SAB2500845),
mouse monoclonal antibody against anti-CD19 (4G7 hybridoma, ATCC),
mouse monoclonal antibody anti-CD3 (OKT3 hybridoma, ATCC),
mouse monoclonal antibody anti-CD4 (Beckman Coulter, #IM0398),
mouse monoclonal antibody anti-CD14 (Beckman Coulter, #A83482),
mouse monoclonal antibody anti-glycophorin A (Beckman Coulter, #IM2210).
rabbit monoclonal antibody against human GAPDH (D16H11, Cell Signaling Technology, #5174)
rabbit polyclonal antibody against human HIF1α (Novus Biologicals, #NB100-134)

Validation

Validation statements provided by manufacturer's website. For anti-GCK and anti-HK2, validation of their specificity by western-blot analysis of cells overexpressing human GCK or human HK2.

## Eukaryotic cell lines

Policy information about cell lines

| | |
|---|---|
| Cell line source(s) | Huh7 were given by M. Binder, Heidelberg,<br>Huh7-GCK were constructed from Huh7 as described in the manuscript |
| Authentication | Genetic characteristics of Huh7 cell line were determined by PCR-single-locus-technology. 21 independent PCRsystems Amelogenin, D3S1358, D1S1656, D6S1043, D13S317, Penta E, D16S539, D18S51, D2S1338, CSF1PO, Penta D, TH01, vWA, D21S11, D7S820, D5S818, TPOX, D8S1179, D12S391, D19S433 and FGA were investigated (Promega, PowerPlex 21 PCR Kit). Certification performed by Eurofins) |
| Mycoplasma contamination | Cell lines were tested negative for mycoplasma contamination by PCR (mycoplasma check, eurofins) |
| Commonly misidentified lines<br>(See ICLAC register) | none |
